# Supplementary material for: Streaming Variational Monte Carlo
Source: IEEE Trans Pattern Anal Mach Intell. Author manuscript; Available in PMC 2023 Apr 9. (PMC10082974; doi:10.1109/TPAMI.2022.3153225)
Supplement: supp1-3153225 [file NIHMS1855392-supplement-supp1-3153225.pdf]

## Appendix A
## Proof that $\hat{p}(y_t|\mathbf{y}_{1:t-1})$ is a consistent estimator for $p(y_t|\mathbf{y}_{1:t-1})$

*Proof.* To prove that $\hat{p}(y_t|\mathbf{y}_{1:t-1})$ is a consistent estimator, we will rely on the delta method [31]. From [60], we know that the central limit theorem (CLT) holds for $\hat{p}(\mathbf{y}_{1:t})$ and $\hat{p}(\mathbf{y}_{1:t-1})$

$$\sqrt{N}(\hat{p}(\mathbf{y}_{1:t-1}) - p(\mathbf{y}_{1:t-1})) \xrightarrow{d} \mathcal{N}(0, \sigma^2_{t-1}), \quad (43)$$
$$\sqrt{N}(\hat{p}(\mathbf{y}_{1:t}) - p(\mathbf{y}_{1:t})) \xrightarrow{d} \mathcal{N}(0, \sigma^2_{t}) \quad (44)$$

where we assume that $\sigma^2_{t-1}$ and $\sigma^2_t$ are finite. We can express $\hat{p}(y_t|\mathbf{y}_{1:t-1})$ as a function of $\hat{p}(\mathbf{y}_{1:t})$ and $\hat{p}(\mathbf{y}_{1:t-1})$,

$$\hat{p}(y_t|\mathbf{y}_{1:t-1}) = g(\hat{p}(\mathbf{y}_{1:t}), \hat{p}(\mathbf{y}_{1:t-1})) = \frac{\hat{p}(\mathbf{y}_{1:t})}{\hat{p}(\mathbf{y}_{1:t-1})}. \quad (45)$$

Since $\frac{p(\mathbf{y}_{1:t})}{p(\mathbf{y}_{1:t-1})} = p(y_t|\mathbf{y}_{1:t-1})$ and $g$ is a continuous function, an application of the Delta method gives

$$\sqrt{N}(\hat{p}(y_t|\mathbf{y}_{1:t-1}) - p(y_t|\mathbf{y}_{1:t-1})) \xrightarrow{d} \mathcal{N}(0, \nabla g^\top \Sigma \nabla g), \quad (46)$$

where $\Sigma_{1,1} = \sigma^2_t$, $\Sigma_{2,2} = \sigma^2_{t-1}$ and $\Sigma_{1,2} = \Sigma_{2,1} = \sigma_{t,t-1}$ where by the Cauchy-Schwartz inequality, $\sigma_{t,t-1}$ is also finite [31]. Thus, as $N \to \infty$, $\hat{p}(y_t|\mathbf{y}_{1:t-1})$ will converge in probability to $p(y_t|\mathbf{y}_{1:t-1})$, proving the consistency of the estimator. □

## Appendix B
## Proof of Theorem 2.1

*Proof.* It is well known that the importance weights produced in a run of SMC are an unbiased estimator of $p(\mathbf{y}_{1:t})$ [21]

$$\mathbb{E}[\hat{p}(\mathbf{y}_{1:t})] = p(\mathbf{y}_{1:t}) \quad (47)$$

where $\hat{p}(\mathbf{y}_{1:t}) = \prod_{j=1}^{t} \frac{1}{N}\sum_{i=1}^{N} w^i_j$. We can apply Jensen's inequality to obtain

$$\log p(\mathbf{y}_{1:t}) \geq \mathbb{E}[\log \hat{p}(\mathbf{y}_{1:t})]. \quad (48)$$

Expanding both sides of (48)

$$\begin{aligned}&\log p(y_t|\mathbf{y}_{1:t-1}) + \log p(\mathbf{y}_{1:t-1})\\ &\quad \geq \mathbb{E}[\log \hat{p}(y_t|\mathbf{y}_{1:t-1})] + \mathbb{E}[\log \hat{p}(\mathbf{y}_{1:t-1})].\end{aligned} \quad (49)$$

Subtracting $\log p(\mathbf{y}_{1:t-1})$ from both sides gives

$$\begin{aligned}&\log p(y_t|\mathbf{y}_{1:t-1})\\ &\quad \geq \mathbb{E}[\log \hat{p}(y_t|\mathbf{y}_{1:t-1})] + \mathbb{E}[\log \hat{p}(\mathbf{y}_{1:t-1})] - \log p(\mathbf{y}_{1:t-1}).\end{aligned} \quad (50)$$

Letting $\mathcal{R}_t(N) = \log p(\mathbf{y}_{1:t-1}) - \mathbb{E}[\log \hat{p}(\mathbf{y}_{1:t-1})]$, where $N$ is the number of samples, we get

$$\log p(y_t|\mathbf{y}_{1:t-1}) \geq \mathbb{E}[\log \hat{p}(y_t|\mathbf{y}_{1:t-1})] - \mathcal{R}_t(N), \quad (51)$$

where by Jensen's inequality (48), $\mathcal{R}_t(N) \geq 0$ for all values of $N$. By the continuous mapping theorem [31],

$$\lim_{N\to\infty} \mathbb{E}[\log \hat{p}(\mathbf{y}_{1:t-1})] = \log p(\mathbf{y}_{1:t-1}). \quad (52)$$

As a consequence, $\lim_{N\to\infty} \mathbb{E}[\mathcal{R}_t(N)] = 0$. By the same logic, and leveraging that $\hat{p}(y_t|\mathbf{y}_{1:t-1})$ is a consistent estimator for $p(y_t|\mathbf{y}_{1:t-1})$, we get that

$$\lim_{N\to\infty} \mathbb{E}[\log \hat{p}(y_t|\mathbf{y}_{1:t-1})] = \log p(y_t|\mathbf{y}_{1:t-1}). \quad (53)$$

Thus $\mathcal{L}_t$ will get arbitrarily close to $\log p(y_t|\mathbf{y}_{1:t-1})$ as $N \to \infty$. □

## Appendix C
## Proof of Corollary 2.1.1

*Proof.* The implicit *smoothing* distribution that arises from performing SMC [25] is defined as

$$\tilde{q}(\mathbf{x}_{1:t}) = p(\mathbf{x}_{1:t}, \mathbf{y}_{1:t})\mathbb{E}\left[\frac{1}{\hat{p}(\mathbf{y}_{1:t})}\right]. \quad (54)$$

We can obtain the implicit filtering distribution by marginalizing out $d\mathbf{x}_{1:t-1}$ from (54)

$$\begin{aligned}&\tilde{q}(x_t|\mathbf{y}_{1:t})\\ &= \int p(\mathbf{x}_{1:t}, \mathbf{y}_{1:t})\mathbb{E}\left[\frac{1}{\hat{p}(\mathbf{y}_{1:t})}\right] d\mathbf{x}_{1:t-1},\\ &= p(x_t, \mathbf{y}_{1:t})\mathbb{E}\left[\frac{1}{\hat{p}(\mathbf{y}_{1:t})}\right],\\ &= p(x_t, y_t|\mathbf{y}_{1:t-1})\mathbb{E}\left[\hat{p}(y_t|\mathbf{y}_{1:t-1})^{-1}\frac{p(\mathbf{y}_{1:t-1})}{\hat{p}(\mathbf{y}_{1:t-1})}\right].\end{aligned} \quad (55)$$

In [25], [39], it was shown that

$$\begin{aligned}\log p(\mathbf{y}_{1:t}) &\geq \mathbb{E}_{q(\mathbf{x}_{1:t})}[\log p(\mathbf{x}_{1:t}, \mathbf{y}_{1:t}) - \log \tilde{q}(\mathbf{x}_{1:t})]\\ &\geq \mathbb{E}[\log \hat{p}(\mathbf{y}_{1:t})].\end{aligned} \quad (56)$$

Rearranging terms in (56), we get

$$\log p(y_t|\mathbf{y}_{1:t-1}) \geq \hat{\mathcal{L}}_t \geq \mathcal{L}_t. \quad (57)$$

where

$$\begin{aligned}\hat{\mathcal{L}}_t &= \mathbb{E}_{q(\mathbf{x}_{1:t})}[\log p(x_t, y_t|\mathbf{y}_{1:t-1}, \mathbf{x}_{1:t-1}) - \log \tilde{q}(x_t|\mathbf{x}_{1:t-1})]\\ &\quad + \mathbb{D}_{\mathrm{KL}}[\tilde{q}(\mathbf{x}_{1:t-1})\|p(\mathbf{x}_{1:t-1}, \mathbf{y}_{1:t-1})] - \log p(\mathbf{y}_{1:t-1}).\end{aligned} \quad (58)$$

By Theorem 2.1, we know that $\lim_{N\to\infty} \mathcal{L}_t = \log p(y_t|\mathbf{y}_{1:t-1})$, and thus

$$\lim_{N\to\infty} \hat{\mathcal{L}}_t = \log p(y_t|\mathbf{y}_{1:t-1}). \quad (59)$$

Leveraging Theorem 1 from [25] we have

$$\lim_{N\to\infty} \mathbb{D}_{\mathrm{KL}}[\tilde{q}(\mathbf{x}_{1:t-1})\|p(\mathbf{x}_{1:t-1}, \mathbf{y}_{1:t-1})] = \log p(\mathbf{y}_{1:t-1}) \quad (60)$$

which implies that

$$\lim_{N\to\infty} \tilde{q}(\mathbf{x}_{1:t-1}) = p(\mathbf{x}_{1:t-1}) \text{ a.e.} \quad (61)$$

thus plugging this into (59)

$$\begin{aligned}&\log p(y_t|\mathbf{y}_{1:t-1})\\ &= \int -\tilde{q}(\mathbf{x}_{1:t}) \log \frac{p(x_t, y_t|\mathbf{y}_{1:t-1}, \mathbf{x}_{1:t-1})}{\tilde{q}(x_t|\mathbf{x}_{1:t-1})} d\mathbf{x}_{1:t}\\ &= \int -\tilde{q}(\mathbf{x}_{1:t}) \log \frac{p(\mathbf{x}_{1:t}|\mathbf{y}_{1:t})p(y_t|\mathbf{y}_{1:t-1})}{\tilde{q}(x_t|\mathbf{x}_{1:t-1})p(\mathbf{x}_{1:t-1}|\mathbf{y}_{1:t-1})} d\mathbf{x}_{1:t}\\ &= \log p(y_t|\mathbf{y}_{1:t-1})\\ &\quad + \int -\tilde{q}(\mathbf{x}_{1:t}) \log \frac{p(x_t|\mathbf{x}_{1:t-1}, \mathbf{y}_{1:t})}{\tilde{q}(x_t|\mathbf{x}_{1:t-1})} d\mathbf{x}_{1:t}\end{aligned} \quad (62)$$

which is true iff $\tilde{q}(x_t|\mathbf{x}_{1:t-1}) = p(x_t|\mathbf{x}_{1:t-1}, \mathbf{y}_{1:t})$ almost everywhere. Thus by Lebesgue's dominated convergence theorem [31]

$$\begin{aligned}&\lim_{N\to\infty} \int \tilde{q}(x_t|\mathbf{x}_{1:t-1}) d\mathbf{x}_{1:t-1}\\ &= \int \lim_{N\to\infty} \tilde{q}(x_t|\mathbf{x}_{1:t-1}) d\mathbf{x}_{1:t-1}\\ &= p(x_t|\mathbf{y}_{1:t}).\end{aligned} \quad (63)$$

□

## Appendix D
## Synthetic NASCAR® Dynamics

An rSLDS [51] with 4 discrete states was used to generate the synthetic NASCAR® track. The linear dynamics for each hidden state were

$$A_1 = \begin{bmatrix} \cos(\theta_1) & -\sin(\theta_1) \\ \sin(\theta_1) & \cos(\theta_1) \end{bmatrix}, \; A_2 = \begin{bmatrix} \cos(\theta_2) & -\sin(\theta_2) \\ \sin(\theta_2) & \cos(\theta_2) \end{bmatrix}, \tag{64}$$

and $A_3 = A_4 = I$. The affine terms were $B_1 = -(A_1 - I)c_1$, ($c_1 = [2, 0]^\top$), $B_2 = -(A_2 - I)c_2$, ($c_2 = [-2, 0]^\top$), $B_3 = [0.1, 0]^\top$ and $B_4 = [-0.35, 0]^\top$. The hyperplanes, $R$, and biases, $r$, were defined as

$$R = \begin{bmatrix} 100 & 0 \\ -100 & 0 \\ 0 & 100 \end{bmatrix}, \quad r = \begin{bmatrix} -200 \\ -200 \\ 0 \end{bmatrix}.$$

A state noise of $Q = 0.001I$ was used.

## Appendix E
## Prediction using SVMC-GP

Let $\tilde{w}_t^i = \frac{w_t^i}{\sum_\ell w_t^\ell}$ be the self-normalized importance weights. At time $t$, given a test point $x_*$ we can approximately sample from the predictive distribution

$$\begin{aligned} p(f_*|x_*, \mathbf{y}_{1:t}) &= \int p(f_*|x_*, \mathbf{z}_t)p(\mathbf{z}_t|\mathbf{y}_{1:t})d\mathbf{z}_t \\ &= \int p(f_*|x_*, \mathbf{z}_t)p(\mathbf{z}_t|\mathbf{x}_{0:t})p(\mathbf{x}_{0:t}|\mathbf{y}_{1:t})d\mathbf{z}_t d\mathbf{x}_{0:t} \\ &= \int p(f_*|x_*, \mathbf{x}_{0:t})p(\mathbf{x}_{0:t}|\mathbf{y}_{1:t})d\mathbf{x}_{0:t} \\ &\approx \sum_{i=1}^N \tilde{w}_t^i p(f_*|x_*, \mathbf{x}_{0:t}^i) \\ &= \sum_{i=1}^N \tilde{w}_t^i \mathcal{N}(v_*^i, \Sigma_*^i) \end{aligned} \tag{65}$$

where

$$v_*^i = m(x_*) + A_*\mu_t^i, \tag{66}$$
$$\Sigma_*^i = C_* + A_*\Gamma_t^i A_*^\top \tag{67}$$

where $A_* = K_{*z}K_{zz}^{-1}$ and $C_* = K_{**} - K_{*z}K_{zz}^{-1}K_{z*} + Q$. The approximate predictive distribution is a mixture of SGPs, allowing for a much more richer approximation to the predictive distribution. Equipped with (65), we approximate the mean of the predictive distribution, $\mu_{f_*}$, as

$$\begin{aligned} \mu_{f_*} &= \int f_* p(f_*|x_*, \mathbf{y}_{1:t})df_* \\ &\approx \int f_* \sum_{i=1}^N \tilde{w}_t^i p(f_*|x_*, \mathbf{x}_{0:t}^i)df_* \\ &= \sum_{i=1}^N \tilde{w}_t^i \int f_* p(f_*|x_*, \mathbf{x}_{0:t}^i)df_* \\ &= \sum_{i=1}^N \tilde{w}_t^i \mathbb{E}_i[f_*] = \sum_{i=1}^N \tilde{w}_t^i v_*^i = \hat{\mu}_{f_*} \end{aligned} \tag{68}$$

where $\mathbb{E}_i[\cdot] = \mathbb{E}_{p(f_*|x_*, \mathbf{x}_{0:t}^i)}[\cdot]$.

Similarly, we can also approximate the covariance of of the predictive distribution, $\Sigma_{f_*}$

$$\begin{aligned} \Sigma_{f_*} &= \int (f_* - \mu_{f_*})(f_* - \mu_{f_*})^\top p(f_*|x_*, \mathbf{y}_{1:t})df_* \\ &\approx \sum_{i=1}^N \tilde{w}_t^i \int (f_* - \mu_{f_*})(f_* - \mu_{f_*})^\top p(f_*|x_*, \mathbf{x}_{0:t}^i)df_* \\ &= \sum_{i=1}^N \tilde{w}_t^i \mathbb{E}_i[(f_* - \mu_{f_*})(f_* - \mu_{f_*})^\top] \\ &= \sum_{i=1}^N \tilde{w}_t^i (\mathbb{E}_i[f_* f_*^\top] - \mathbb{E}_i[f_*]\mu_{f_*}^\top - \mu_{f_*}\mathbb{E}_i[f_*]^\top + \mu_{f_*}\mu_{f_*}^\top) \\ &= \sum_{i=1}^N \tilde{w}_t^i (\Sigma_*^i + v_*^i v_*^{i\top} - v_*^i \mu_{f_*}^\top - \mu_{f_*} v_*^{i\top} + \mu_{f_*}\mu_{f_*}^\top) \\ &\approx \sum_{i=1}^N \tilde{w}_t^i (\Sigma_*^i + v_*^i v_*^{i\top} - v_*^i \hat{\mu}_{f_*}^\top - \hat{\mu}_{f_*} v_*^{i\top} + \hat{\mu}_{f_*}\hat{\mu}_{f_*}^\top). \end{aligned} \tag{69}$$

## Appendix F
## Winner-Take-All Spiking Neural Network

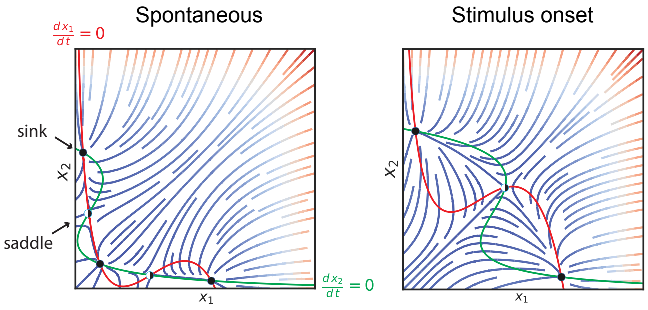


Figure 6. Mean field reduction of the Winner-Take-All spiking neural network.

In Figure 6 the mean-field reduction of the spiking network model is shown [58].
